# Myeloid SENP3 deficiency protects mice from diet and age-induced obesity via regulation of YAP1 SUMOylation

**DOI:** 10.1007/s00018-023-05050-w

**Published:** 2023-12-09

**Authors:** Yangjing Jiang, Min Liang, Long Chen, Jian Wang, Yijie Huang, Huanhuan Huo, Danrui Xiao, Yunwen Hu, Zi Wang, Qingqi Ji, Yanjie Li, Zhaohua Cai, Ben He

**Affiliations:** Department of Cardiology, Shanghai Chest Hospital, Shanghai Jiao Tong University School of Medicine, Shanghai, 200030 China

**Keywords:** Adipose tissue, Obesity, Macrophage, SENP3, SUMOylation, YAP1

## Abstract

Obesity is characterized by chronic low-grade inflammation, which is driven by macrophage infiltration in adipose tissue and leads to elevated cytokines such as interleukin-1β (IL-1β) in the circulation and tissues. Previous studies demonstrate that SENP3, a redox-sensitive SUMO2/3-specific protease, is strongly implicated in the development and progression of cancer and cardiovascular diseases. However, the role of SENP3 in obesity-associated inflammation remains largely unknown. To better understand the effects of SENP3 on adipose tissue macrophage (ATM) activation and function within the context of obesity, we generated mice with myeloid-specific deletion of SENP3 (*Senp3*^*flox/flox*^;*Lyz2-Cre* mice). We found that the expression of SENP3 is dramatically increased in ATMs during high-fat diet (HFD)-induced obesity in mice. *Senp3*^*flox/flox*^;*Lyz2-Cre* mice show lower body weight gain and reduced adiposity and adipocyte size after challenged with HFD and during aging. Myeloid-specific SENP3 deletion attenuates macrophage infiltration in adipose tissue and reduces serum levels of inflammatory factors during diet and age-induced obesity. Furthermore, we found that SENP3 knockout markedly inhibits cytokine release from macrophage after lipopolysaccharide and palmitic acid treatment in vitro. Mechanistically, in cultured peritoneal macrophages, SENP3 protein level is enhanced by IL-1β, in parallel with the upregulation of Yes-associated protein 1 (YAP1). Moreover, we demonstrated that SENP3 modulates de-SUMO modification of YAP1 and SENP3 deletion abolishes the upregulation of YAP1 induced by IL-1β. Most importantly, SENP3 deficiency reduces YAP1 protein level in adipose tissue during obesity. Our results highlight the important role of SENP3 in ATM inflammation and diet and age-induced obesity.

**Supplementary Information:**

The online version contains supplementary material available at 10.1007/s00018-023-05050-w.

## Introduction

Obesity is an increasingly prevalent metabolic disease characterized by excessive fat accumulation especially excess of white adipose tissue (WAT). Evidence suggests that many obesity comorbidities, such as type 2 diabetes mellitus, steatohepatitis, and cardiovascular diseases, are related to obesity-induced chronic low-grade inflammation [1, 2]. Macrophages are the primary immune cells involved in obesity-associated inflammation in both mice and humans [3, 4]. Emerging evidence suggests that ATMs have additional roles beyond classical M1/M2 polarization in obesity and related comorbidities [5, 6]. Despite recent advances in the study of ATMs, the underlying mechanism by which macrophages are activated in adipose tissues and how they promote obesity and related comorbidities has not yet been fully elucidated.

SUMOylation is a reversible posttranslational modification (PTM) that covalently conjugates Small Ubiquitin-like Modifier (SUMO) to target proteins and modulates the protein level, subcellular localization, and transcriptional activity of the modified proteins [7–10]. The SUMO proteases (SENPs) de-SUMOylate modified proteins and thus are critical for maintaining the SUMOylation level of substrates required for normal physiology [11–13]. SENP3 has been demonstrated to play important roles in the development and progression of cancer and cardiovascular diseases, which was mediated by its de-SUMOylation of various protein substrates [9, 14–17]. We previously demonstrated that SENP3 regulates vascular smooth muscle cell function and vascular remodeling via de-SUMOylation of β-catenin and regulation of its stability [9]. However, the role of SENP3 in macrophage activation and function in the context of obesity has never been investigated.

Yes-associated protein (YAP) and transcriptional coactivator with PDZ-binding motif (TAZ) (thereafter YAP/TAZ) are the main downstream effectors of the Hippo signaling pathway. Extensive studies have established the role of the Hippo pathway components in growth, development, and cancer biology. Emerging evidence suggests that they are also involved in regulating inflammatory responses and inflammation-associated diseases [18–20]. For example, Liu et al. reported the regulation and function of macrophage YAP in the context of atherosclerosis [18]. YAP/TAZ modulates macrophage polarization and plays important roles in cardiac repair after myocardial infarction [19]. Moreover, YAP aggravates inflammatory bowel disease by regulating M1/M2 macrophage polarization. The expression and transcriptional activity of YAP1 are widely regulated at the transcriptional, post-transcriptional, and post-translational levels. It has been well demonstrated that YAP1 can be regulated by PTMs including phosphorylation and ubiquitination [18, 21, 22]. Whether YAP1 is modified and regulated by SUMOylation remains to be fully elucidated.

In this present study, we assessed the expression and function of macrophage SENP3 in adipose tissue during obesity; we also examined the SUMO modification of YAP1. We demonstrated that SENP3 accumulates in ATMs in obese adipose tissue. Myeloid-specific SENP3 deletion reduces adiposity, adipocyte size, and ATM infiltration in adipose tissue in the context of diet and age-induced obesity. Mechanistically, SENP3 deSUMOylates YAP1 and regulates its protein level. These findings suggest that SENP3 is essential for regulating ATMs activation and obesity-related inflammation.

## Materials and methods

### Mice

*Senp3*^*flox/flox*^ mice were kindly provided by Prof. Jing Yi (Shanghai Jiao Tong University School of Medicine, Shanghai, China). *Lyz2-Cre* transgenic mice (Stock No. NM-KI-215037) and C57BL/6 mice were purchased from Shanghai Model Organisms Center Inc (Shanghai, China). *Senp3*^*flox/flox*^ mice were intercrossed with *Lyz2-Cre* mice to generate *Senp3*^*flox/flox*^;*Lyz2-Cre* mice. All mouse experimental protocols were approved by the Institutional Animal Care and Use Committee at Shanghai Chest Hospital affiliated to Shanghai Jiao Tong University and followed relevant ethical regulations.

C57BL/6 mice, *Senp3*^*flox/flox*^ control mice, and Senp3^*flox/flox*^*;Lyz2-Cre* mice were fed either chow diet or high-fat diet (HFD, 60% kcal fat, #12492, Research diets Inc, NJ, USA) starting at 8 weeks of age for 16 weeks or 24 weeks. Body weight was measured every 4 weeks until mice were sacrificed. Another cohort of *Senp3*^*flox/flox*^*;Lyz2-Cre* mice and *Senp3*^*flox/flox*^ littermates aged 18-month-old and fed a chow diet throughout the study was used for age-induced obesity mouse model.

### Tissue collection, processing, and histology

Mice were euthanized by inhalation of 5% isoflurane and cervical dislocation where appropriate. Tissue sections for pathological diagnosis or immunofluorescence were fixed in 10% neutral buffered formalin and embedded in paraffin or optimal cutting temperature (OCT) compound. Paraffin-embedded sections or OCT-embedded sections were cut at 5 μm or 8 μm thickness, respectively. Paraffin-embedded tissue sections were stained with hematoxylin and eosin (H & E) according to standard protocols [9, 23]. All images were recorded using an Olympus digital camera (Tokyo, Japan).

### Immunohistochemistry

For immunohistochemical staining, paraffin-embedded tissue sections were deparaffinized, rehydrated, and subjected to antigen retrieval. Endogenous peroxidase activity was blocked using 0.3% H_2_O_2_ for 20 min. Tissue sections were blocked with normal goat serum (Biogenex, HK112-9K) and incubated with primary antibodies against Senp3 (Cell Signaling Technology [CST]; Cat# 5591; 1:100) and YAP1 (CST, Cat# 14074, 1:100). Goat antirabbit/mouse IgG (DAKO, Cat# K4061, ready-to-use) were used as secondary antibodies. The reaction was visualized using DAB (DAKO, Cat# K3468) and sections were counterstained with hematoxylin. All images were recorded using an Olympus digital camera (Tokyo, Japan).

### Isolation and culture of murine peritoneal macrophages

Peritoneal macrophages were isolated as follows. Mice were injected intraperitoneally with 1 ml 4% thioglycolate broth (Sigma; Cat# T9032). After 3 days, mice were euthanized, and peritoneal macrophages were harvested using 10 ml icecold PBS. Cells were cultured in RPMI-1640 (Hyclone, Logan, UT, USA) supplemented with 10% fetal bovine serum (FBS), 100 U/ml penicillin, and 100 U/ml streptomycin.

### Western blotting

Total protein was prepared from mouse adipose tissue, and western blotting was performed as briefly described below. Proteins were quantified with a Pierce BCA Protein Assay Kit, separated by SDS-PAGE, and transferred to nitrocellulose membranes. Membranes were blocked with 5% nonfat milk dissolved in Tris-buffered saline with Tween 20 (TBST) at 37 °C for 1 h, and incubated with primary antibodies against SENP3 (CST; Cat# 5591; 1:1000), iNOS (Abcam; Cat# ab3523; 1:1000), IκBα (CST; Cat# 4812; 1:1000), Phospho-IκBα (Ser32) (CST; Cat# 2859; 1:1000), YAP1 (CST; Cat# 14074; 1:1000), phospho-YAP (Ser127) (CST; Cat# 4911; 1:1000), Flag (Sigma; Cat# F1804; 1:5000), HA (Abcam; Cat# ab9110; 1:4000), β-actin (Santa cruz, Cat# sc47778, 1:1000), and GAPDH (Santa Cruz, Cat# sc32233, 1:1000) at 4 °C overnight. After incubation with horseradish peroxidase-conjugated secondary antibodies at 37 °C for 1 h, proteins were detected using Pierce ECL Western Blotting Substrate and quantified using Quantity One 4.4.0 software (Bio-Rad, Hercules, CA, USA).

### RNA extraction and quantitative (real-time) PCR (qPCR)

Total RNA was extracted from primary mouse peritoneal macrophages or mouse adipose tissues using Trizol reagent (Invitrogen), according to the manufacturer’s instructions. Total RNA (1 μg) was reverse-transcribed into first-strand cDNA, and qPCR amplification was performed using iQ SYBR Green Supermix (Bio-Rad, Cat# 1708882) and the CFX96 Touch™ Real-time PCR Detection System (Bio-Rad). The primer sequences used are presented in Supplementary Table 1. Relative mRNA expression was calculated using the comparative ΔΔCT method and the resulting values were normalized to *18S* ribosomal RNA expression. PCR was performed in triplicate for each experiment. The results presented represent three independent experiments.

### Enzyme-linked immunosorbent assay (ELISA)

Serum IL-1β, IL-6, and TNFα concentrations were measured with enzyme-linked immunosorbent assay (ELISA) kits (Abcam, Cat# ab197742, ab46100, and ab46105, respectively), according to the manufacturer's instructions.

### Statistical analysis

Statistical analyses were performed using GraphPad 8 (https://www.graphpad.com/). Unpaired two-tailed Student’s *t* tests were used to calculate significant differences between two groups. Multiple comparison correction analysis was performed using one-way ANOVA with Tukey’s post hoc HSD test. *p* < 0.05 was considered statistically significant.

## Results

### SENP3 is highly expressed in ATMs during high-fat diet-induced obesity

**Fig. 1:**
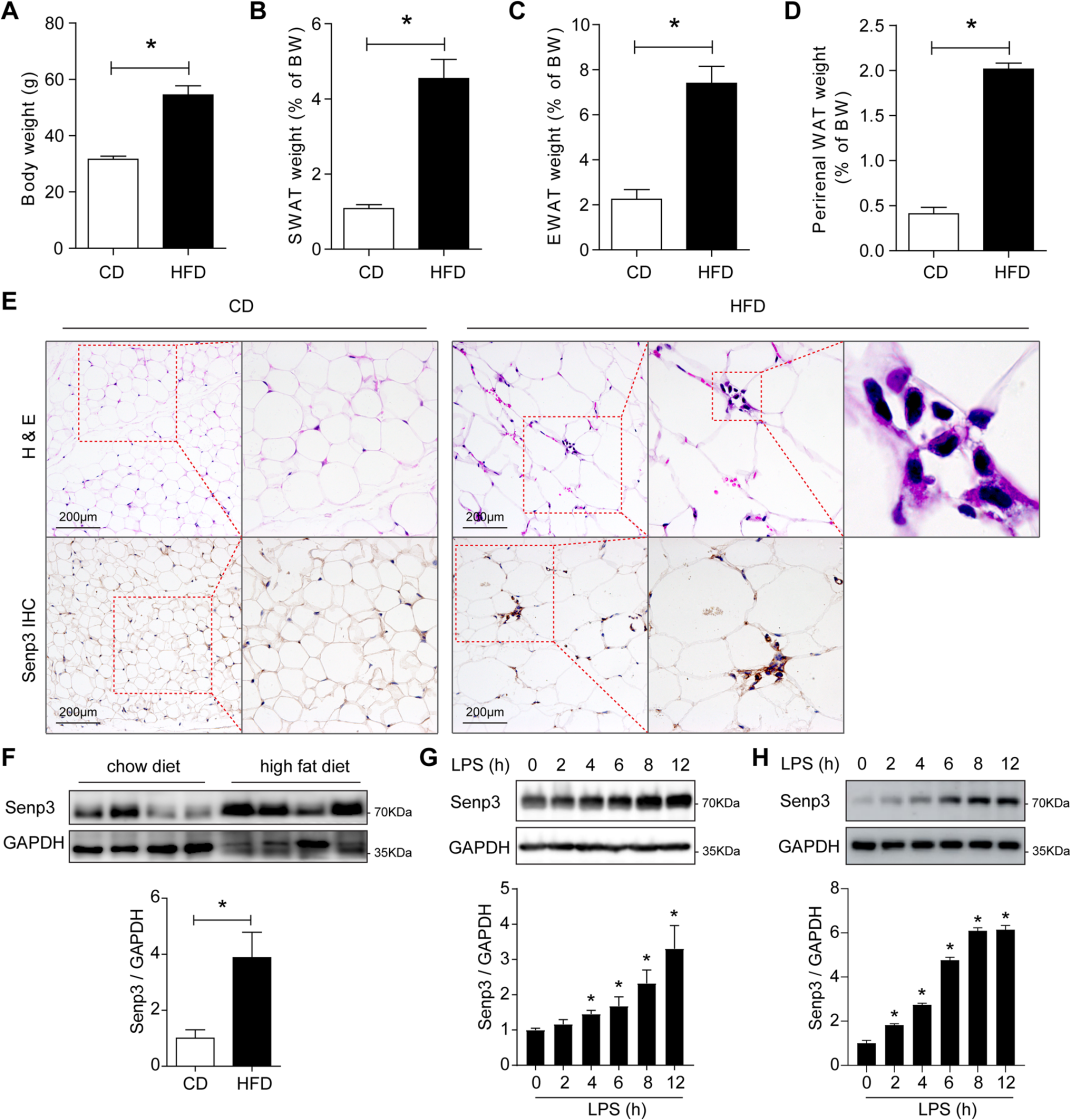
SENP3 is highly expressed in adipose tissue macrophage during high-fat diet-induced obesity. **A–D** Body weight and absolute weights of subcutaneous white adipose tissue (sWAT), epididymal white adipose tissue (eWAT), and perirenal white adipose tissue (perirenal WAT) in C57BL/6 mice fed either a normal chow diet (CD) or high-fat diet (HFD) for 16 weeks. Data represent mean ± SEM (*n* = 5 per group). **E** H & E staining and immunohistochemical staining of SENP3 for eWAT sections from C57BL/6 mice fed either a CD or HFD for 16 weeks. **F** Western blot analysis of SENP3 protein expression in eWAT from C57BL/6 mice fed a HFD compared with control mice fed a CD. Data represent mean ± SEM (*n* = 4 biological replicates per group). **G**, **H** Primary mouse peritoneal macrophages (**G**) and Raw 264.7 cells (**H**) were treated with LPS (100 ng/ml) for the indicated time points. Protein expression of SENP3 was examined by western blotting (*n* = 3, *p < 0.05). *p* values were determined using student’s *t*-test (**A** and **F**) or one-way ANOVA with Tukey’s multiple comparisons test (**G**, **H**). For all panels, *p < 0.05

To investigate the role of SENP3 in ATM function during obesity, we generated the mouse model of high-fat diet (HFD)-induced obesity and examined the expression of SENP3 in white adipose tissue. We found that body weight and the relative tissue weights of subcutaneous white adipose tissue (sWAT), epididymal white adipose tissue (eWAT), and perirenal white adipose tissue (perirenal WAT) were significantly increased after HFD treatment (Fig. 1A–D). HFD induced formation of the crown-like structures (CLSs) composed of inflammatory macrophages surrounding adipocytes in eWAT (Fig. 1E), which is consistent with the previous studies [24, 25]. Interestingly, we found that SENP3 expression was dramatically enhanced in the area of CLSs which are indicative of ATMs in eWAT from HFD-fed mice as compared to control mice (Fig. 1E). Moreover, western blotting analysis revealed the upregulation of SENP3 in eWAT from HFD mice (Fig. 1F). In addition, we found that SENP3 was upregulated time dependently after exposure to lipopolysaccharide (LPS) treatment in primary cultured peritoneal macrophages (Fig. 1G) and Raw 264.7 cell line (Fig. 1H). Taken together, all these results suggest a potential role of macrophage SENP3 in the development of HFD-induced obesity.

### Myeloid SENP3 deficiency protects mice from HFD-induced obesity and systemic inflammation

**Fig. 2:**
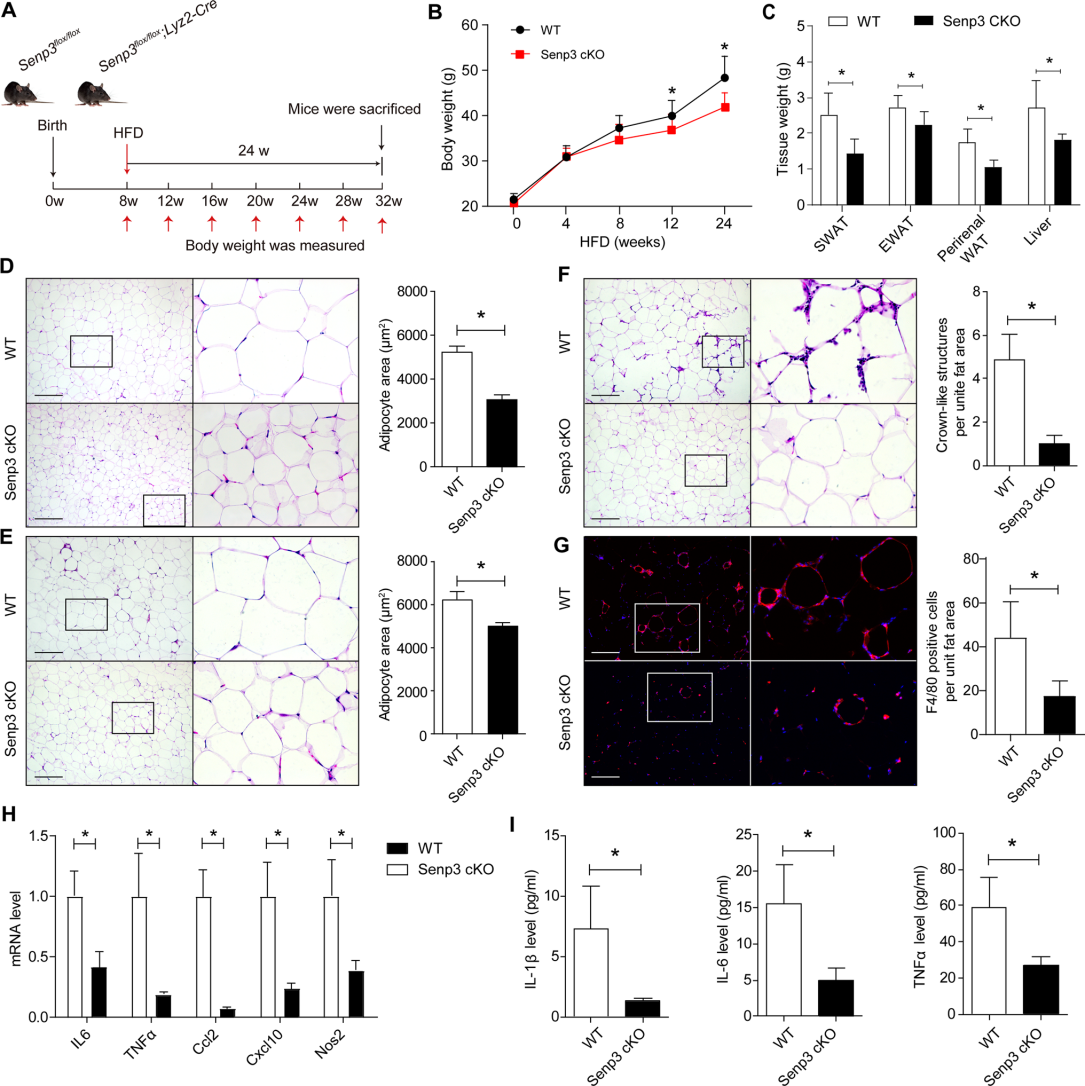
Myeloid SENP3 deficiency protects mice from high-fat diet-induced obesity and systemic inflammation. **A** A schematic illustration of the experimental protocol. **B**, **C** Body weight and absolute weights of subcutaneous white adipose tissue (sWAT), epididymal white adipose tissue (eWAT), perirenal white adipose tissue (perirenal WAT), and liver in *Senp3*^*flox/flox*^*;Lyz2-Cre* (Senp3 cKO) mice and wild-type (WT) *Senp3*^*flox/flox*^ littermates fed a high-fat diet (HFD) for 24 weeks. Data represent mean ± SEM (*n* = 9 for WT mice and *n* = 13–14 for Senp3 cKO mice. **D**, **E** Representative images of H & E staining and quantification of adipocyte area for sWAT (**D**) and eWAT (**E**) in WT and Senp3 cKO mice fed a HFD for 24 weeks. Data represent mean ± SEM (*n* = 8–10 per group). **F** Representative images of H & E staining and quantification of crown-like structure per unit fat area for eWAT in WT and Senp3 cKO mice fed a HFD for 24 weeks. Data represent mean ± SEM (*n* = 9 per group). **G** Representative images of immunofluorescent staining of F4/80 (red) and quantification of F4/80 positive cells per unit fat area for eWAT in WT and Senp3 cKO mice fed a HFD for 24 weeks. Blue indicates DAPI staining of nuclei. Data represent mean ± SEM (*n* = 5 per group). **H** The mRNA levels of the indicated genes were assessed by qRT-PCR for eWAT in WT and Senp3 cKO mice fed a HFD for 24 weeks. Data represent mean ± SEM (*n* = 12 biological replicates per group). **I** Serum levels of IL-1β, IL-6, and TNFα were measured by ELISA. Data represent mean ± SEM (*n* = 8 for WT mice and *n* = 12–13 for Senp3 cKO mice. *p* values were determined using student’s *t*-test (**B**–**I**). For all panels, *p < 0.05. The boxed areas in the left panels are shown at a higher magnification in the right panels (**D**–**G**). Scale Bar: 200 μm

To investigate the functional importance of SENP3 expressed by macrophage during obesity, we generated the mice with myeloid-specific SENP3 deletion by intercrossing *Senp3*^*flox/flox*^ mice with *Lyz2-Cre* mice (Fig. S1A). In *Senp3*^*flox/flox*^;*Lyz2-Cre* mice, SENP3 was ablated in primary peritoneal macrophage, rather than in heart, liver, spleen, and kidney (Fig. S1B). We found that when challenged with HFD (Fig. 2A), *Senp3*^*flox/flox*^;*Lyz2-Cre* mice gained lower body weight than their wild-type (WT) *Senp3*^*flox/flox*^ littermates (Fig. 2B). In addition, *Senp3*^*flox/flox*^;*Lyz2-Cre* mice showed much reduced weights of various types of WAT, including sWAT, eWAT, and perirenal WAT (Fig. 2C). Moreover, HFD-fed *Senp3*^*flox/flox*^;*Lyz2-Cre* mice showed a significant reduction in liver weight, compared with *Senp3*^*flox/flox*^ littermates (Fig. 2C). Accordingly, H & E staining of sWAT and eWAT sections revealed significantly decreased adipocyte size in *Senp3*^*flox/flox*^;*Lyz2-Cre* mice, when compared with *Senp3*^*flox/flox*^ littermates (Fig. 2D and E). Therefore, SENP3 deficiency in macrophage protects mice against diet-induced obesity.

As macrophages are the primary immune cells involved in obesity-associated inflammation [4, 26, 27], we check the CLSs in eWAT of both WT and *Senp3*^*flox/flox*^; *Lyz2-Cre* mice and found that *Senp3*^*flox/flox*^; *Lyz2-Cre* mice had much reduced HFD-induced CLS formation (Fig. 2F). This was further confirmed by the immunofluorescent staining of F4/80, marker for macrophage, which showed that eWAT of *Senp3*^*flox/flox*^;*Lyz2-Cre* mice was infiltrated by less macrophages compared with *Senp3*^*flox/flox*^ littermates (Fig. 2G). This observation was further validated by quantitative real-time PCR, indicating that *Senp3*^*flox/flox*^; *Lyz2-Cre* mice exhibited less expression of cytokines and inflammatory factors in eWAT (Fig. 2H)*.* It is well established that cytokines and inflammatory factors secreted by immune cells (especially macrophages) ignite localized and systemic inflammation during diet-induced obesity. We therefore checked the level of inflammatory factors in mouse serum and found that serum IL-1β, IL-6, and TNFα were found to be dramatically decreased in *Senp3*^*flox/flox*^; *Lyz2-Cre* mice (Fig. 2I). These findings indicate that in response to HFD, myeloid SENP3 deficiency mitigates local and systemic inflammation in mice.

### Myeloid SENP3 deficiency protects mice from age-induced obesity and systemic inflammation

**Fig. 3:**
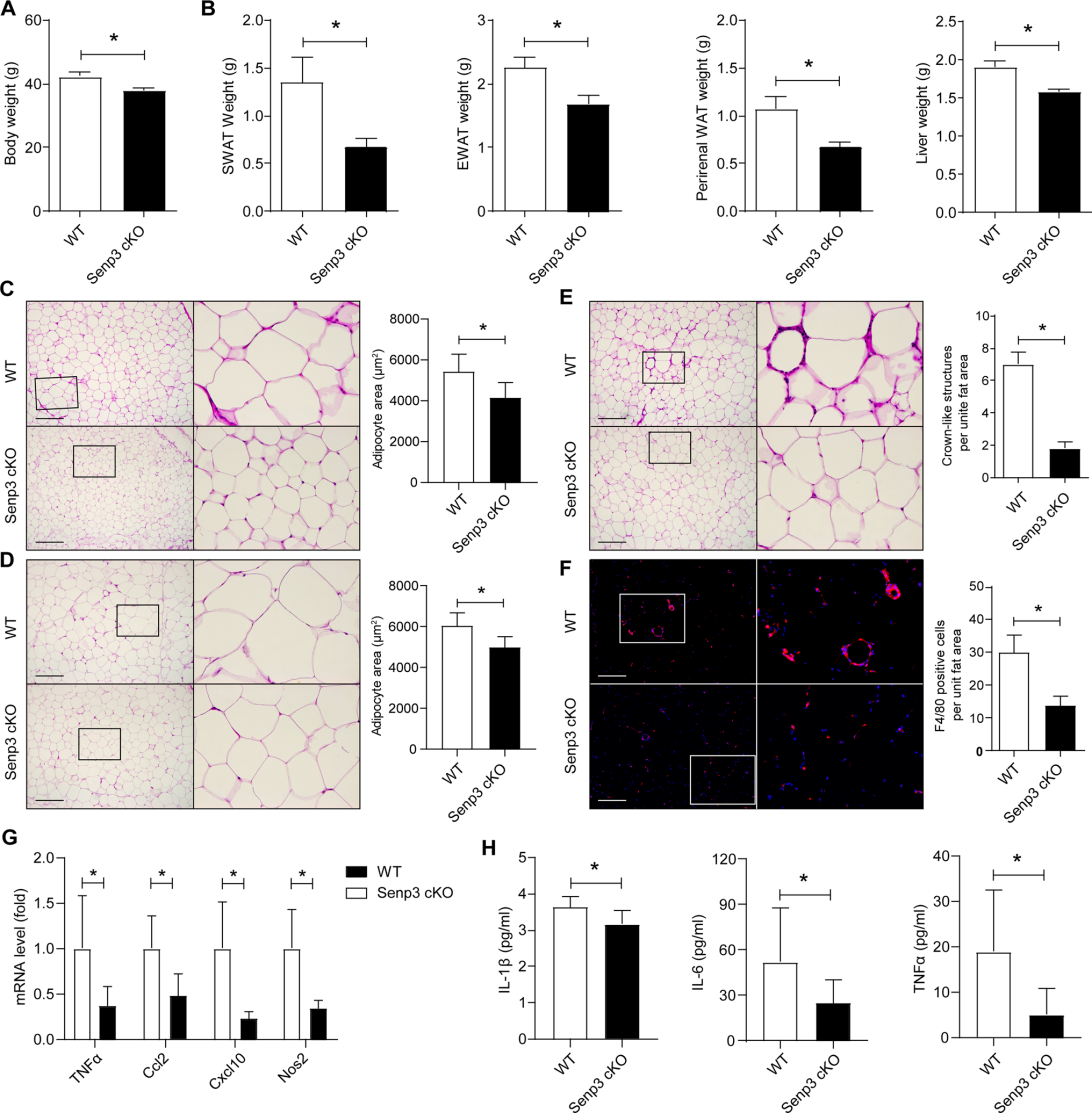
Myeloid SENP3 deficiency mitigates age-induced obesity and systemic inflammation in mice. **A**, **B** Body weight and absolute weights of subcutaneous white adipose tissue (sWAT), epididymal white adipose tissue (eWAT), perirenal white adipose tissue (perirenal WAT), and liver in *Senp3*^*flox/flox*^*;Lyz2-Cre* (Senp3 cKO) mice and wild-type (WT) *Senp3*^*flox/flox*^ littermates aged 18-month-old. Data represent mean ± SEM (*n* = 7 for WT mice and *n* = 10 for Senp3 cKO mice. **C**, **D** Representative images of H & E staining and quantification of adipocyte area for sWAT (C) and eWAT (D) in WT and Senp3 cKO mice aged 18-month-old. Data represent mean ± SEM (*n* = 5–6 for WT mice and *n* = 8–10 for Senp3 cKO mice. **E** Representative images of H & E staining and quantification of crown-like structure per unit fat area for eWAT in WT and Senp3 cKO mice aged 18-month-old. Data represent mean ± SEM (*n* = 5 for WT mice and *n* = 8 for Senp3 cKO mice. **F** Representative images of immunofluorescent staining of F4/80 (red) and quantification of F4/80 positive cells per unit fat area for eWAT in WT and Senp3 cKO mice aged 18-month-old. Blue indicates DAPI staining of nuclei. Data represent mean ± SEM (*n* = 5 per group). **G** The mRNA levels of the indicated genes were assessed by qRT-PCR for eWAT in WT and Senp3 cKO mice aged 18-month-old. Data represent mean ± SEM (*n* = 6 per group). **H** Serum levels of IL-1β, IL-6, and TNFα were measured by ELISA. Data represent mean ± SEM (*n* = 7 for WT mice and *n* = 9–10 for Senp3 cKO mice. *p* values were determined using student’s *t*-test (**A**–**H**). For all panels, *p < 0.05. The boxed areas in the left panels are shown at a higher magnification in the right panels (**C**–**F**). Scale Bar: 200 μm

Adipose tissue undergoes dramatic and unique changes in mass and distribution during aging [28]. Increased fat mass accumulation and fat redistribution are commonly observed in aging populations [29, 30]. To further investigate the role of SENP3 expressed by macrophage in age-induced obesity, we followed up *Senp3*^*flox/flox*^; *Lyz2-Cre* mice and *Senp3*^*flox/flox*^ littermates until they are almost 18 month old. We found that the body weight of aged *Senp3*^*flox/flox*^; *Lyz2-Cre* mice was dramatically decreased when compared with aged *Senp3*^*flox/flox*^ littermates (Fig. 3A). Adipose depots at different anatomic locations from *Senp3*^*flox/flox*^; *Lyz2-Cre* and *Senp3*^*flox/flox*^ littermates aged 18 months were surgically extracted. We found that the relative tissue weights of sWAT, eWAT, perirenal WAT, and liver were significantly decreased in *Senp3*^*flox/flox*^; *Lyz2-Cre* mice (Fig. 3B). However, there were no significant changes for the relative tissue weights of heart, lung, and kidney (data not shown). Accordingly, histological analysis further confirmed the significant decreased in adipocyte size in sWAT and eWAT of aged *Senp3*^*flox/flox*^; *Lyz2-Cre* mice, compared with aged *Senp3*^*flox/flox*^ littermates (Fig. 3C and D).

We further examined the inflammatory status in aged *Senp3*^*flox/flox*^; *Lyz2-Cre* mice and *Senp3*^*flox/flox*^ littermates. We found that myeloid-specific SENP3 deficiency inhibits adipose tissue inflammation during aging, as shown by the decreased number of CLSs and F4/80-positive macrophage infiltration in eWAT (Fig. 3E and F). The results of quantitative real-time PCR further confirmed a significant reduction of cytokine release in eWAT in *Senp3*^*flox/flox*^; *Lyz2-Cre* mice (Fig. 3G). Consistently, we found that myeloid-specific SENP3 deletion reduces serum levels of inflammatory factors during age-induced obesity (Fig. 3H). Taken together, all these results suggest SENP3 promotes ATM accumulation in adipose tissue and systemic inflammation during obesity.

### SENP3 deficiency mitigates cytokine or inflammatory factors release from macrophage

**Fig. 4:**
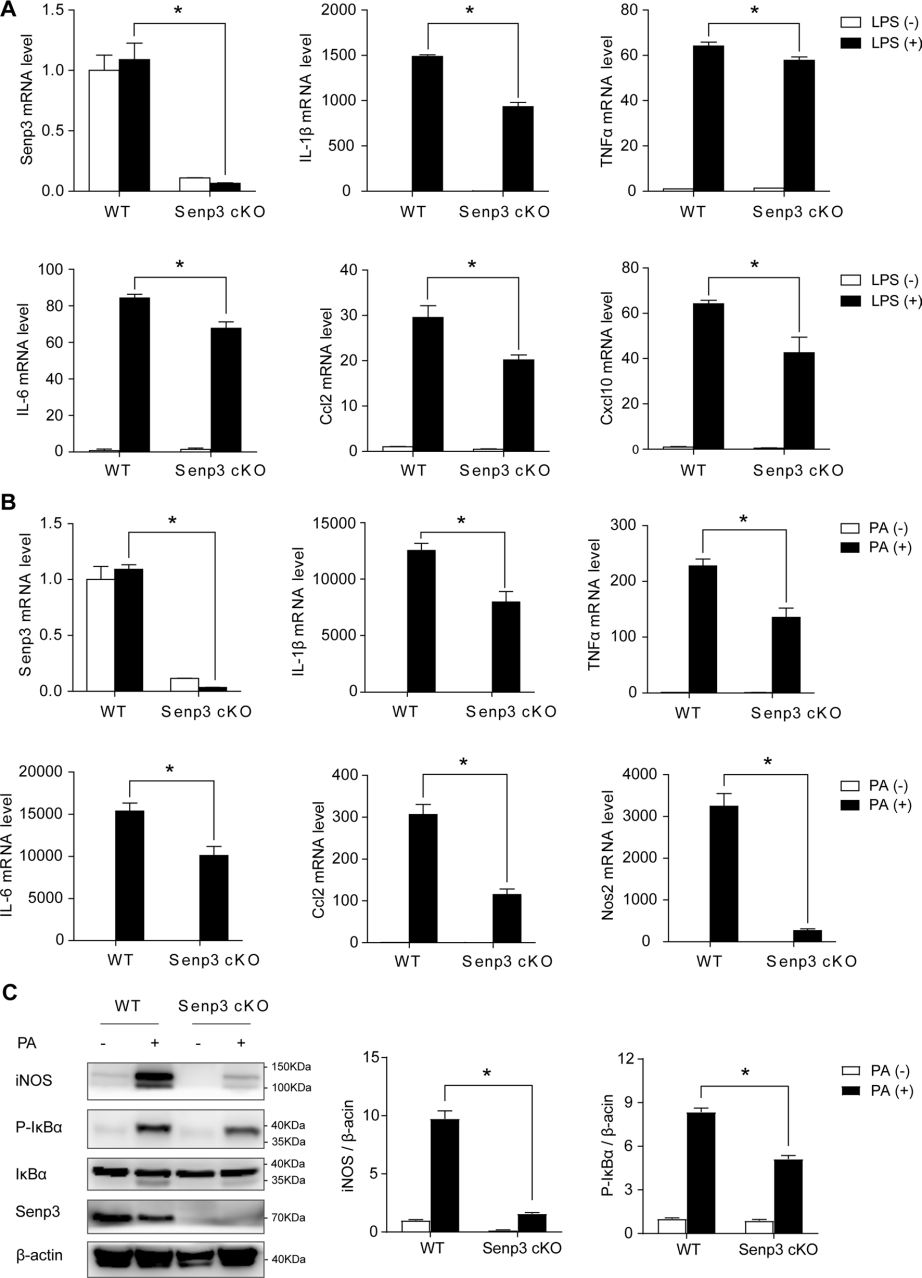
SENP3 deficiency mitigates cytokine or inflammatory factors release from macrophages. **A** Primary mouse peritoneal macrophages isolated from WT and *Senp3*^*flox/flox*^*;Lyz2-Cre* (Senp3 cKO) mice were incubated with LPS (100 ng/ml) for 24 h. The mRNA levels of proinflammatory cytokines IL-1β, TNFα, IL-6, Ccl2, and Cxcl10 were assessed by qRT-PCR. Data represent mean ± SEM (*n* = 3 biological replicates per group). **B** Primary mouse peritoneal macrophages isolated from WT and Senp3 cKO mice were incubated with PA (500 μM) for 24 h. The mRNA levels of proinflammatory cytokines IL-1β, TNFα, IL-6, Ccl2, and Nos2 were assessed by qRT-PCR. Data represent mean ± SEM (*n* = 3 biological replicates per group). **C** Primary mouse peritoneal macrophages isolated from WT and Senp3 cKO mice were incubated with PA (500 μM) for 24 h. Protein expression of iNOS, IκBα, P-IκBα, and SENP3 was examined by western blotting (*n* = 3, *p < 0.05). *p* values were determined using student’s *t*-test (**A**–**C**). For all panels, *p < 0.05

To establish the functional significance of SENP3, we examined the effect of SENP3 knockout on macrophage cytokine release. We first examined the expression of the major inflammatory cytokines in primary peritoneal macrophages from WT and *Senp3*^*flox/flox*^;*Lyz2-Cre* mice exposed to 100 ng/ml LPS for 24 h. The results of quantitative real-time PCR showed that the mRNA transcriptional levels of IL-1β, TNFα, IL-6, Ccl2, and Cxcl10 were significantly decreased in macrophages from *Senp3*^*flox/flox*^;*Lyz2-Cre* mice compared with those from WT mice (Fig. 4A). Moreover, we further examined the expression of inflammatory cytokines in WT and SENP3 knockout peritoneal macrophages exposed to palmitic acid (PA). The results showed that the mRNA transcriptional levels of IL-1β, TNFα, IL-6, Ccl2, and Nos2 were significantly decreased in macrophages from *Senp3*^*flox/flox*^;*Lyz2-Cre* mice when compared with those from WT mice (Fig. 4B). This result was further conformed by western blotting, showing the decreased expression of iNOS and P-IκBα in SENP3 knockout macrophages (Fig. 4C). These results indicate that SENP3 plays critical roles in inflammation and cytokine production in macrophages.

### SENP3 de-SUMOylates YAP1 and regulates its protein level

**Fig. 5:**
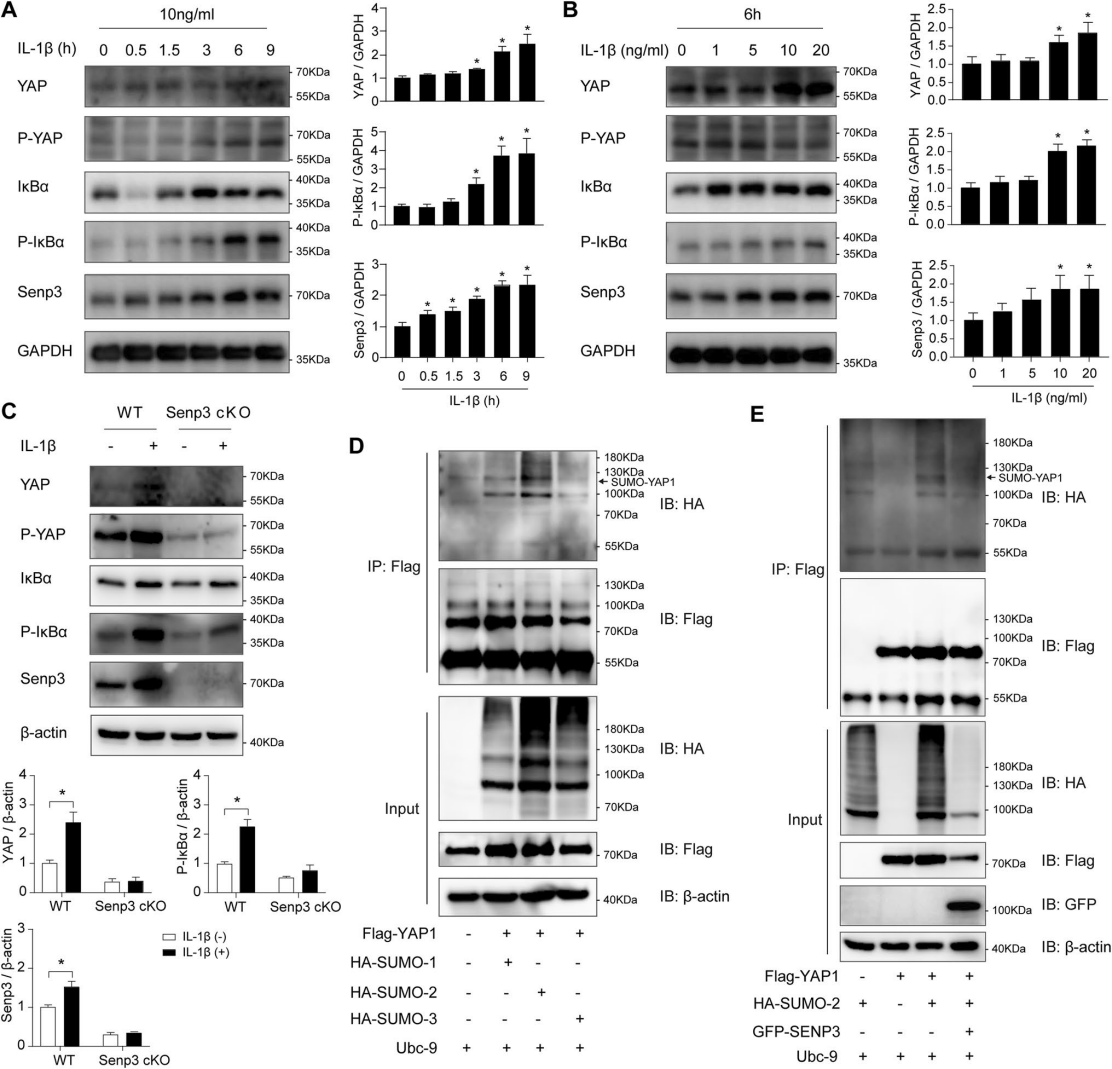
SENP3 de-SUMOylates YAP1 and regulates its protein level. **A** Raw264.7 cells were treated with IL-1β (10 ng/ml) for the indicated time points. Protein expression of YAP1, P-YAP1, IκBα, P-IκBα, and SENP3 was examined by western blotting. The relative protein level was quantified for YAP1, P-IκBα, and SENP3 (*n* = 3, *p < 0.05). **B** Raw264.7 cells were treated with increasing concentrations of IL-1β for 6 h. Protein expression of YAP1, P-YAP1, IκBα, P-IκBα, and SENP3 was examined by western blotting. The relative protein level was quantified for YAP1, P-IκBα, and SENP3 (*n* = 3, *p < 0.05). **C** Primary mouse peritoneal macrophages from WT and *Senp3*^*flox/flox*^*;Lyz2-Cre* (Senp3 cKO) mice were treated with IL-1β (10 ng/ml) for 8 h. Protein expression of YAP1, P-YAP1, IκBα, P-IκBα, and SENP3 was examined by western blotting. The relative protein level was quantified for YAP1, P-IκBα, and SENP3 (*n* = 3, *p < 0.05). **D** 293T cells were transfected with Flag-YAP1, HA-SUMO-1, HA-SUMO-2, or HA-SUMO-3, and Ubc-9 for 24 h. The SUMOylation of Flag-YAP1 was determined by IP assay using Flag-beads and western blotting using anti-Flag, anti-HA, and anti-β-actin antibodies. **E** 293T cells were transfected with Flag-YAP1, HA-SUMO-2, GFP-SENP3, and Ubc-9 for 24 h. The SUMOylation of Flag-YAP1 was determined by IP assay using Flag-beads and western blotting using anti-Flag, anti-HA, anti-GFP, and anti-β-actin antibodies. *p* values were determined using one-way ANOVA with Tukey’s multiple comparisons test (**A**, **B**) or student’s *t*-test (**C**). For all panels, *p < 0.05

Growing evidences have indicated that YAP1 signaling is involved in the regulation macrophage function and inflammation [18–20]. To define the possible mechanism by which SENP3 exerts its effect on macrophage function and inflammation, we first examined the level of SENP3 and YAP1 signaling after inflammatory factor IL-1β treatment. Immunoblotting demonstrated that IL-1β markedly increased SENP3 protein expression in a time- and dose-dependent manner, which is in parallel with time-dependent and dose-dependent upregulation of YAP1 (Fig. 5A and B). Most importantly, we found that SENP3 deletion abolished the upregulation of YAP1 induced by IL-1β (Fig. 5C). Meanwhile, the activation of IκBα signaling was attenuated after SENP3 deletion (Fig. 5C).

As SENP3 acts as a SUMO2/3-specific protease, we speculated that YAP1 can be SUMOylated and SENP3 can de-SUMOylate YAP1. We therefore first predicted the SUMOylation possibility of YAP1 using computational system-based software including SUMOsp 2.0 and SUMOplot™. One lysine residue for YAP1 was consistent in the results of SUMOsp 2.0 and SUMOplot™ and highly conserved among different species (data not shown). As shown in Fig. 5D, YAP1 SUMOylation was detected by immunoprecipitation in 293T cells co-expressed with Flag-YAP1 and HA-SUMO-1, 2, 3. Moreover, the result showed that SENP3 deconjugated SUMO-2 from YAP1 (Fig. 5E). As cysteine 532 of SENP3 is responsible for its enzymatic activity [31], we further overexpressed an enzymatically inactive mutant SENP3 (SENP3-C532A) and found that SENP3 deconjugated SUMO-2 from Flag-YAP1, while SENP3-C532A mutant that lost de-SUMOylating activity did not change the SUMOylation of Flag-YAP1 (Fig. S3). These results suggest that SENP3 de-SUMOylates YAP1 and regulates its protein level.

### Myeloid-specific SENP3 deficiency attenuates YAP1 signaling in mouse WAT during obesity

**Fig. 6:**
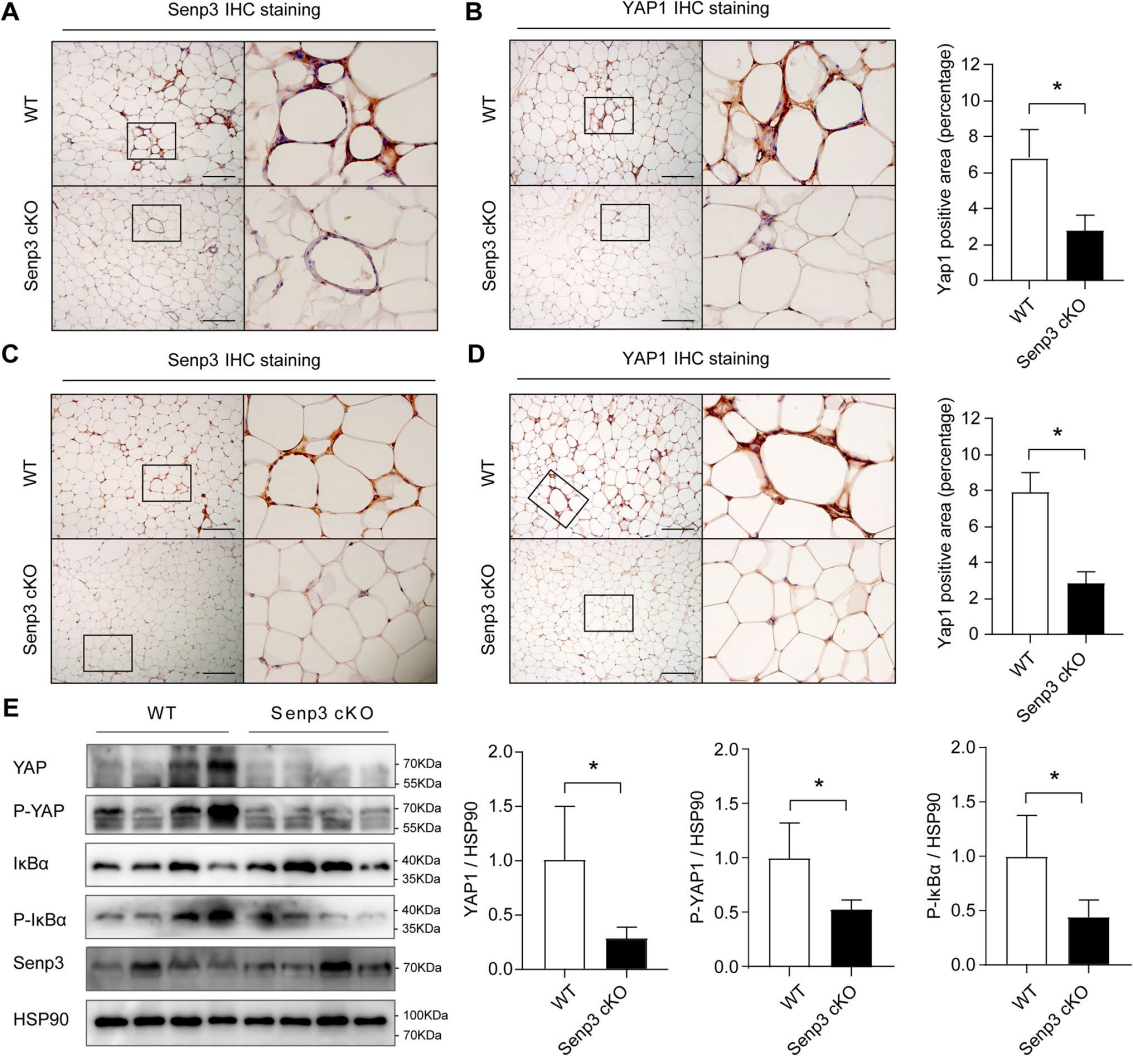
Myeloid-specific SENP3 deficiency attenuates YAP1 signaling in mouse WAT during diet and age-induced obesity. **A** Representative images of immunohistochemistry staining of SENP3 in WT and *Senp3*^*flox/flox*^*;Lyz2-Cre* (Senp3 cKO) mice fed a high-fat diet (HFD) for 24 weeks. **B** Representative images of immunohistochemistry staining of YAP1 and quantification of percentage of YAP1-positive area in WT and Senp3 cKO mice fed a HFD for 24 weeks. Data represent mean ± SEM (*n* = 5 per group). **C** Representative images of immunohistochemistry staining of SENP3 in WT and Senp3 cKO mice aged 18-month-old. **D** Representative images of immunohistochemistry staining of YAP1 and quantification of percentage of YAP1-positive area in WT and Senp3 cKO mice aged 18-month-old. Data represent mean ± SEM (*n* = 5 per group). **E** Western blot analysis of protein expression for YAP1, P-YAP1, IκBα, P-IκBα, and SENP3 in eWAT from WT and Senp3 cKO mice fed a HFD for 24 weeks (*n* = 4, *p < 0.05). *p* values were determined using student’s *t*-test (**B**, **D**, **E**). For all panels, *p < 0.05. The boxed areas in the left panels are shown at a higher magnification in the right panels (**A**–**D**). Scale Bar: 200 μm

To further define the in vivo role of SENP3 in YAP1 signaling and obesity, we investigated the in vivo expression of SENP3 and YAP1 in adipose tissue of *Senp3*^*flox/flox*^; *Lyz2-Cre* mice and *Senp3*^*flox/flox*^ littermates. Immunohistochemistry staining of SENP3 confirmed that the expression of SENP3 was decreased in ATMs from HFD-fed *Senp3*^flox/flox^; *Lyz2-Cre* mice when compared with HFD-fed *Senp3*^*flox/flox*^ littermates (Fig. 6A). Most importantly, the in vivo expression of YAP1 in ATMs was markedly decreased in the eWAT from *Senp3*^*flox/flox*^;*Lyz2-Cre* mice compared with *Senp3*^*flox/flox*^ littermates (Fig. 6B). This result was further confirmed in aged *Senp3*^*flox/flox*^;*Lyz2-Cre* mice and *Senp3*^*flox/flox*^ littermates (Fig. 6C and D). Western blotting analysis further demonstrated the dramatically decreased YAP1 signaling in *Senp3*^*flox/flox*^; *Lyz2-Cre* mice compared with *Senp3*^*flox/flox*^ littermates, accompanied by the attenuated IκBα signaling (Fig. 6E). These results suggest that YAP1 is the important substrate for SENP3 which plays important roles in macrophage function.

## Discussion

Mounting studies have demonstrated that macrophages are recruited into adipose tissue and secrete inflammatory cytokines such as IL-1β and TNF-α during obesity, thereby causing local and systemic inflammation. Although these central roles of macrophage-secreted inflammatory cytokines in the development of obesity and related comorbidities have been well established, the precise molecular mechanism underlying how ATMs are activated in obese adipose tissue has not been fully elucidated. In this present study, we highlight the critical role of SUMO-specific protease 3 (SENP3) in the pathogenesis of obesity and obesity-associated inflammation. We found that SENP3 protein levels were markedly increased after exposure to associated stimuli relevant to obesity, both in vivo and in vitro. In addition, myeloid-specific SENP3 deletion attenuated HFD and age-induced obesity and systemic inflammation, which was partially mediated by YAP1 SUMOylation change.

SUMO specific proteases (SENPs) are cysteine proteases which play important roles in maintaining the balance between SUMO/de-SUMOylated proteins required for normal cellular physiology. There are 6 isoforms of SENPs identified in humans (including SENP1–3 and 5–7) [32]. Previous studies have highlighted the pivotal role of SENPs in the development of various diseases, including cancers and cardiovascular diseases [9, 14–16, 33–35]. Moreover, an increasing evidence has demonstrated the involvement of SENPs in the regulation of macrophage function and inflammation. For example, Lao et al. reported that SENP3 potentiates LPS-induced TLR4 signaling via deSUMOylation of MKK7 and myeloid-specific SENP3 deletion attenuates LPS-induced endotoxin shock [36] and acute lung injury [37]. However, there are few studies concerning the role of SENPs in ATM activation and adipose tissue inflammation in obesity. To our knowledge, our study is the first study that demonstrates the important role of SENP3, a SUMO-specific protease, in the development of diet and age-induced obesity. In this present study, *Senp3*^*flox/flox*^; *Lyz2-Cre* mice exhibited reduced HFD and age-induced adiposity, adipocyte size, and ATM infiltration, which leads to attenuated systemic inflammation. Our findings demonstrate that SENP3 is essential for ATMs activation and function during the context of obesity.

The Hippo-YAP/TAZ pathway is an important signaling pathway that regulates organ size and tissue homeostasis and has been strongly implicated in the progression of various cancers and cardiovascular diseases [38]. Emerging studies have revealed the critical pathophysiological roles of the YAP pathway in inflammation and macrophage function [18, 19]. In the present study, we demonstrated that YAP1 is modified by SUMOylation which can be reversibly de-conjugated by SENP3 in 293T cells. However, we failed to show the endogenous SUMOylation bands in macrophages, possibly due to the antibody problem. Nevertheless, we found that SENP3 protein level was enhanced by IL-1β in macrophage, in parallel with the upregulation of YAP1. SENP3 deletion abolished the upregulation of YAP1 induced by IL-1β. However, as degradation of YAP1 was well demonstrated to be regulated by phosphorylation, whether the SUMO/de-SUMOylation status of YAP1 affects YAP1 protein level directly or indirectly via affecting its phosphorylation status is unknown.

In summary, we provided the first evidence that SENP3 accumulates in ATMs of adipose tissue during diet and age-induced obesity, which promotes macrophage infiltration in adipose tissue and systemic inflammation via regulation of YAP1 SUMOylation. This work might help for increased understanding of adipose tissue inflammation induced by obesity and may ultimately lead to new approaches to prevent and treat obesity. Further work is needed to fully understand the exact role of SENP3 and SENP3-mediated SUMO/de-SUMOylation status of specific protein substrates in ATM during obesity and related comorbidities.

### Supplementary Information

Below is the link to the electronic supplementary material.

Supplementary file1 (DOCX 337 KB)

## Data Availability

The data that support the findings of this study are available from the corresponding author on reasonable request.
