# Supplementary material for: Myeloid SENP3 deficiency protects mice from diet and age-induced obesity via regulation of YAP1 SUMOylation
Source: Cell Mol Life Sci. 2023 Dec 9;81(1):4. doi: 10.1007/s00018-023-05050-w (PMC10710392; doi:10.1007/s00018-023-05050-w)
Supplement: Supplementary file 1 — Supplementary file1 (DOCX 337 KB) [file 18_2023_5050_MOESM1_ESM.docx]

**Supplemental data**


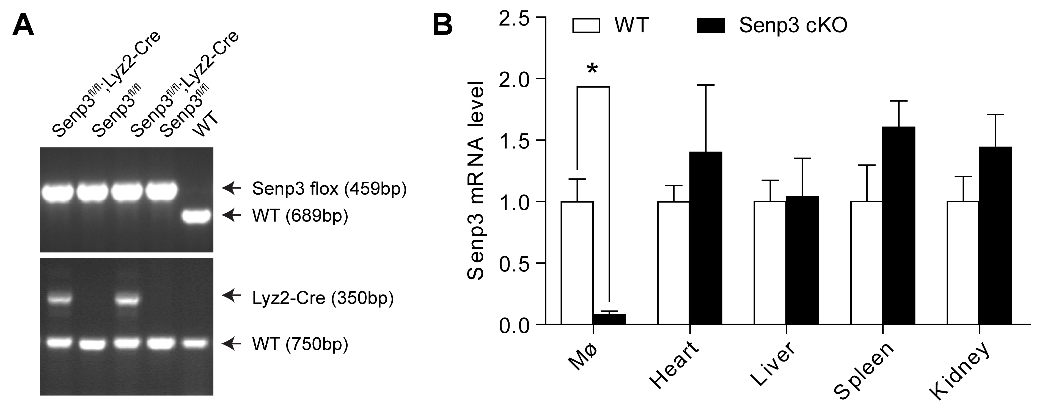


**Fig. S1.**  **Generation of mice with myeloid-specific SENP3 deletion by intercrossing Senp3^flox/flox^ mice with *Lyz2-Cre* mice.** **(A)** Genotype analysis. **(B)** The mRNA level of Senp3 was assessed by qRT-PCR for peritoneal macrophage (mø), heart, liver, spleen, and kidney from *Senp3^flox/flox^;Lyz2-Cre* (Senp3 cKO) mice and wild-type (WT) Senp3*^flox/flox^* littermates (n=3, **p*<0.05).


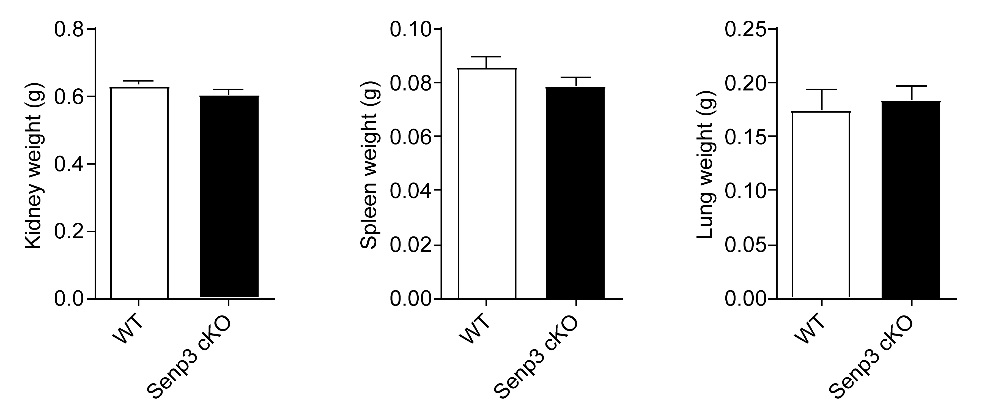


**Fig. S2.** Absolute weights of kidney, spleen, and liver in Wild type (WT) and *Senp3^flox/flox^;Lyz2-Cre* (Senp3 cKO) mice aged 18-month-old. Data represent mean ± SEM (n = 5-7 for WT mice and n=9 for Senp3 cKO mice. P values were determined using student’s t-test. *p < 0.05.


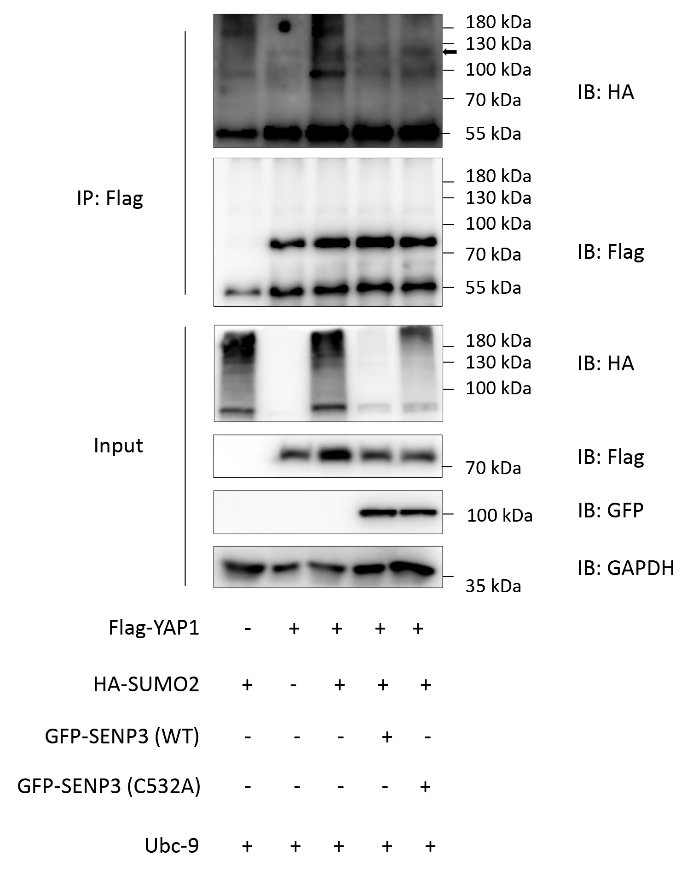


**Fig. S3.**  **SENP3 deconjugates SUMO-2 from Flag-YAP1, while SENP3-C532A mutant does not change the SUMOylation of Flag-YAP1.** 293T cells were transfected with Flag-YAP1, HA-SUMO-2, GFP-SENP3-WT/GFP-SENP3-C532A, and Ubc-9 for 24h. The SUMOylation of Flag-YAP1 was determined by IP assay using Flag-beads and western blotting using anti-Flag, anti-HA, anti-GFP, and anti-GAPDH antibodies. The arrowhead indicates the modified SUMO-YAP1 bands.

**Supplementary Table 1. The primer sequences for qPCR in this study**

| Genes |  | Sequence (5’-3’) |
| --- | --- | --- |
| 18S | Forward | GTCTGTGATGCCCTTAGATG |
| 18S | Reverse | AGCTTATGACCCGCACTTAC |
| Mouse Senp3 | Forward | ACTCCCAGCGAACTCTAA |
| Mouse Senp3 | Reverse | TAATACAAAGGCACCACA |
| Mouse IL-1β | Forward | CTCAATGGACAGAATATCAACCAACA |
| Mouse IL-1β | Reverse | ACAGGACAGGTATAGATTCTTTCCTTTG |
| Mouse IL-6 | Forward | TACCACTTCACAAGTCGGAGG |
| Mouse IL-6 | Reverse | GCTATGGTACTCCAGAAGACC |
| Mouse TNFα | Forward | GTTCTATGGCCCAGACCCTCACA |
| Mouse TNFα | Reverse | TACCAGGGTTTGAGCTCAGC |
| Mouse Ccl2 | Forward | TAAAAACCTGGATCGGAACCAAA |
| Mouse Ccl2 | Reverse | GCATTAGCTTCAGATTTACGGGT |
| Mouse Cxcl10 | Forward | CCAAGTGCTGCCGTCATTTTC |
| Mouse Cxcl10 | Reverse | GGCTCGCAGGGATGATTTCAA |
| Mouse Nos2 | Forward | GTTCTCAGCCCAACAATACAAGA |
| Mouse Nos2 | Reverse | GTGGACGGGTCGATGTCAC |
